# Darunavir-based dual therapy in HIV experienced patients

**DOI:** 10.7448/IAS.17.4.19782

**Published:** 2014-11-02

**Authors:** Gaetana Sterrantino, Mauro Zaccarelli, Antonio Di Biagio, Andrea Rosi, Bianca Bruzzone, Paola Cicconi, Tiziana Carli, Maria Luisa Biondi, Andrea Antinori, Dario Bartolozzi, Giovanni Penco

**Affiliations:** 1 Malattie Infettive e Tropicali, Azienda Ospedaliera Universitaria Careggi, Florence, Italy; 2 Unità Operativa Immunodeficienze Virali, INMI L. Spallanzani IRCCS, Rome, Italy; 3 Malattie Infettive e Tropicali, Azienda Ospedaliera Universitaria San Martino, Genoa, Italy; 4 Dipartimento di biotecnologie mediche, Università di Siena, Siena, Italy; 5 Malattie Infettive e Tropicali, Azienda Ospedaliera San Paolo, Milan, Italy; 6 Malattie Infettive e Tropicali, Ospedale Misericordia, Grosseto, Italy; 7 Diagnostica Molecolare Infettivologica, Azienda Ospedaliera San Paolo, Milan, Italy; 8 Dipartimento Clinico, INMI L. Spallanzani IRCCS, Rome, Italy; 9 Malattie Infettive, Ente Ospedaliero Ospedali Galliera, Genoa, Italy

## Abstract

**Background:**

We assessed the virological response of DRV/r-based dual therapy in drug-experienced patients included in the Italian antiretroviral resistance database (ARCA).

**Materials and Methods:**

Patients included in the study were treated with DRV/r in association with raltegravir (RAL), etravirine (ETV) or maraviroc (MAR) following treatment failure(s) and with a resistance test and at least one follow-up visit available. Observation was censored at last visit under dual therapy and survival analysis and proportional hazard models were used, taking virological failure (confirmed >50 c/mL HIV-RNA) as the end-point.

**Results:**

Of the total 221 patients included, 149 (67.4%) started DRV/r with RAL, 45 (20.4%) with ETV, 27 (12.2%) with MAR. Patients characteristics at the start of dual regimen were as follows: mean number of previous regimens, nine (IQR: 5–13); non-B subtype, 17 (7.7%); median CD4 count, 347 (IQR: 246–544); undetectable viral load, 74 (33.5%). Full DRV/r resistance was detected in one (0.5%, HIV-DB interpretation system), 13 (5.9%, ANRS) and 17 patients (7.7%, Rega). 69 virological failures (31.2%) were observed during follow-up. At survival analysis, the overall proportion of failure was 29.2% at one year and 33.8% at two years. The proportion of failure was lower in patients starting with undetectable versus detectable viral load (13.3% and 25.2% versus 37.4% and 38.8% at one and two years, respectively, p=0.001 for both analyses) and in patients treated with DRV 600 BID versus 800 QD (HR: 0, 56; 95% CI 0.31–0.99; p<0.05). By regimen, patients treated with DRV/r-RAL showed a non-significant lower proportion of failure (27.7% at one year, 32.0% at two years) if compared with DRV/r-MAR (35.9%, 47.1%) and DRV/r-ETV (34.1%, 34.1% at one and two years). In the adjusted proportional model, no significant difference among the three regimens was detected. A significant lower risk of failure was associated with higher overall GSS (HIV-DB HR: 0.53, 95% CI 0.32–0.88, p=0.014; Rega 0.60, 0.40-0.88, p<0.01; ANRS 0.55, 0.34–0.90, p=0.017), while a higher risk of failure was associated with detectable HIV-RNA (3.02, 1.70–5.72, p<0.001).

**Conclusions:**

Among experienced patients, the best candidates to dual-therapy regimens including DRV/r are those with undetectable viral load and higher GSS. The association with RAL is the most commonly used but no clear advantage with respect to ETV or MAR was observed in our dataset, possibly due to the limited sample size.

**Table 1:** Probability of virological failure in experienced patients treated with DRV/r-based Dual Therapy

	HR	IC 95% Lower	IC 95% Upper	p value
Male Gender	0.905	0.497	1.648	0.745
Age (per year)	1.021	0.994	1.049	0.127
IDU	0.759	0.429	1.343	0.343
Non Italian	1.390	0.485	3.983	0.540
HIV non-subtype vs B	1.979	0.858	4.561	0.109
HCV positivity	1.070	0.981	1.168	0.128
N Previous Regimens	0.993	0.897	1.100	0.890
N PI used	1.062	0.928	1.214	0.382
Und VL at Dual switch	3.022	1.696	5.720	<0.001
Baseline CD4	0.973	0.920	1.030	0.348
CD4 nadir	1.032	0.900	1.183	0.653
Raltegravir	1			
MAR (vs RAL)	1.372	0.673	2.797	0.385
ETV (vs RAL)	1.157	0.473	2.831	0.750
DRV 600 bid (vs 800 QD)	0.556	0.314	0.986	<0.05
GSS (REGA, each point)	0.595	0.400	0.884	<0.01

